# Association of *Xmn* I Polymorphism and Hemoglobin E Haplotypes on Postnatal Gamma Globin Gene Expression in Homozygous Hemoglobin E

**DOI:** 10.1155/2012/528075

**Published:** 2012-09-19

**Authors:** Supachai Ekwattanakit, Yuwarat Monteerarat, Suchada Riolueang, Kalaya Tachavanich, Vip Viprakasit

**Affiliations:** ^1^Graduate Program and Medical Scholar Program in Immunology, Department of Immunology, Faculty of Medicine Siriraj Hospital, Mahidol University, 2 Prannok Road, Bangkok Noi, Bangkok 10700, Thailand; ^2^Thalassemia Center, Faculty of Medicine Siriraj Hospital, Mahidol University, 2 Prannok Road, Bangkok Noi, Bangkok 10700, Thailand; ^3^Hematology/Oncology Division, Department of Pediatrics, Faculty of Medicine Siriraj Hospital, Mahidol University, 2 Prannok Road, Bangkok Noi, Bangkok 10700, Thailand

## Abstract

*Background and Objectives*. To explore the role of *cis*-regulatory sequences within the **β** globin gene cluster at chromosome 11 on human **γ** globin gene expression related to Hb E allele, we analyze baseline hematological data and Hb F values together with **β** globin haplotypes in homozygous Hb E. *Patients and Methods*. 80 individuals with molecularly confirmed homozygous Hb E were analyzed for the **β** globin haplotypes and *Xmn* I polymorphism using PCR-RFLPs. 74 individuals with complete laboratory data were further studied for association analyses. *Results*. Eight different **β** globin haplotypes were found linked to Hb E alleles; three major haplotypes were (a) (III), (b) (V), and (c) (IV) accounting for 94% of Hb E chromosomes. A new haplotype (Th-1) was identified and most likely converted from the major ones. The majority of individuals had Hb F < 5%; only 10.8% of homozygous Hb E had high Hb F (average 10.5%, range 5.8–14.3%). No association was found on a specific haplotype or *Xmn* I in these individuals with high Hb F, measured by alkaline denaturation. Conclusion. The cis-regulation of **γ** globin gene expression might not be apparent under a milder condition with lesser globin imbalance such as homozygous Hb E.

## 1. Introduction

Beside producing abnormal variant, hemoglobin E (HbE), the G→A substitution in codon 26 (Glu→Lys) of the *β*-globin gene (*β* ^E^) could also produce *β* ^+^ thalassemia due to decreased functional HbE-mRNA, secondary to alternative splicing mechanism [1]. However, the clinical phenotype in homozygous Hb E (Hb EE) is rather asymptomatic with very mild anemia. In contrast, patients with HbE/*β* thalassemia have a more diverse clinical phenotype from transfusion dependent to very mild disease [2–5]. Although, understanding of clinical phenotypic diversity in patients with Hb E/*β* thalassemia has long been a topic of several investigations, at present, the genotype-phenotype correlation of this so-called single gene disorder remains obscure. 

Variation of postnatal *γ* globin expression and HbF production in these patients was thought to be one of the main genetic factors responsible for clinical heterogeneity found in Hb E/*β* thalassemia by reducing globin imbalance and ameliorating ineffective erythropoiesis. Through erythroid development, the *γ* globin expression was regulated by interactions between *cis*-acting sequences within the *β* globin cluster and *trans*-acting factors such as BCL-11A, cMYB, and TOX [1, 6–8]. The most significant genetic factor in* cis* associated with high HbF is *Xmn* I polymorphism located at −158 upstream to the ^G^ *γ* globin genes [9]. In a recent study using a more refined SNP analysis of the *β* globin gene cluster in HbE/*β* thalassemia has shown that there was no other variant elsewhere which has a comparable level of association with that of *Xmn* I site and the T allele (*Xmn* I, +) was almost always in *cis* with the HbE alleles [10]. To further explore the role of *cis*-acting sequences on Hb F production under the less hematopoietic stress and globin chain imbalance in Hb E disorders, we analyzed 7 known single nucleotide polymorphisms within the 50 kb of the *β* globin gene cluster to construct Hb E-linked *β* globin haplotypes together with *Xmn* I polymorphism. The *β* globin haplotypes of Hb E have been characterized previously in Thailand and within the region of Southeast Asia but they were principally utilized to identify origin, spread, and anthropology of different ethnic groups found with Hb E [11–13]. However there were no study to correlate such findings with postnatal production of Hb F. Recently, we have extensively analyzed up to 76 Thai individuals with Hb E [14] and identified several cases with significantly high Hb F levels in their steady stage and several of them had persistently high Hb F during the follow-up period. Therefore, these individuals provide a different model to explore the role of the *β* globin haplotypes and *Xmn* I (representing *cis*-regulatory sequences) on propensity of the *γ* globin expression. Moreover the association analysis between Hb E alleles and persisting Hb F production in our Thai population might be useful to confirm the findings and apply in a more clinical significant syndrome such as Hb E/*β* thalassemia.

## 2. Materials and Methods

Ethylene diamine tetraacetic acid (EDTA) blood was obtained from 80 Thai individuals with homozygous HbE after informed consent. They were parents, siblings, and unrelated individuals who were tested due to microcytic anemia at Department of Pediatrics, Faculty of Medicine Siriraj Hospital as a part of an ongoing project studying natural history and genotype-phenotype correlation in Hb E disorders in Thailand. This study was approved by a local ethical committee at Mahidol University. Complete hematological and hemoglobin studies have been performed using standard techniques as described previously [14]. Quantification of Hb E and Hb F % was performed by calculation from cellulose acetate electrophoresis and for Hb F, it was subsequently confirmed by alkaline denaturation method [15].

### 2.1. Molecular Diagnosis of Homozygous Hb E by PCR-Restriction Fragment Length Polymorphism (RFLP)

**Figure 1:**
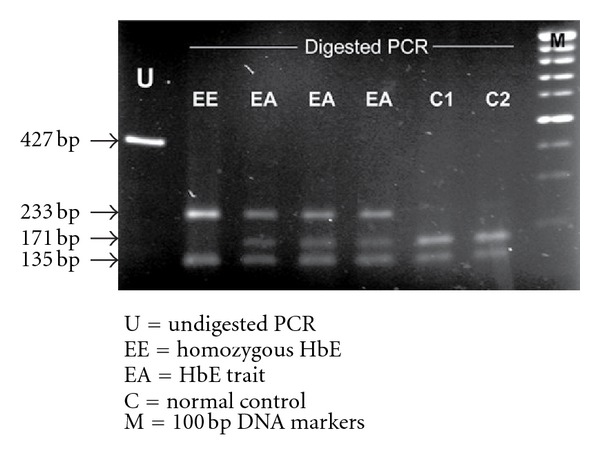
Molecular analysis of HbE mutation using PCR-RFLP by *Mnl* I digestion.

Genomic DNA was extracted using the standard phenol/chloroform procedure. Genotype of homozygous Hb E was confirmed in every case at the molecular level. PCR amplified a fragment of 427 bp in which it contains two *Mnl *I restriction sites, one at the 5′end used as an internal control for digestion (135 bp) and another one linked to CD26 (171 and 62 bp). Subsequently we digested the PCR products using *Mnl *I using the conditions recommended by the manufacturer (New England Biolabs, Beverly, MA); Hb E mutation (G→A) abolishes this *Mnl* I site therefore digestion demonstrated the 233 bp and 135 bp (internal control) (as shown in Figure 1). Further details of this molecular testing have been described elsewhere [14].

### 2.2. *β* Globin Gene Haplotypes and Frameworks by PCR-RFLPs

PCR amplifications were carried out by using the pairs of primers under the optimal conditions as previously described [16]. PCR-based RFLPs covering 7 enzymatic restriction sites across 50 kb of the *β*-globin gene cluster were amplified from the regions *ε*, ^G^ *γ*, ^A^ *γ*, 5′*φβ*, 3′*φβ*, 5′*β*, and 3′*β* and were subsequently digested with *Hin*dII, *Hin*dIII, *Hin*dIII, *Hin*dII, *Hin*dII, *Ava*II, and *Hin*fI, respectively, to identify nucleotide substitutions within and around the *β* globin gene cluster [16]. Haplotypes and frameworks were designated based on the presence (+) or absence (−) of all these restriction sites; two PCR-RFLP sites of the *Ava*II in the intron-2 of the *β* globin gene and of the *Bam*HI located 3′ to the *β* globin gene were used to identify the frameworks. In addition, the *Xmn* I polymorphic site was genotyped by the same technique. The haplotype analysis was performed by using the Phase-Standard-analysis version 2.1.2 [17, 18] to construct Hb E-linked haplotypes. Haplotypes with a low frequency (<0.5%) were excluded from further analyses.

### 2.3. Statistical Analysis

SPSS statistical software package Version 10.0 (SPSS Inc., 2000) was used for data analysis. Paired and unpaired *t*-tests were used to determine the differences between groups. *P *values (*P*) less than 0.05 were considered to be statistically significant.

## 3. Results

From 80 individuals with homozygous Hb E, we could successfully genotypes all 8 polymorphic sites in 77 cases (154 chromosomes of Hb E, Table 1). These individuals were mainly from Bangkok and the Central plain of Thailand. Eight different *β* globin haplotypes associated with HbE alleles have been constructed with two novel ones, (− − − − − + −) and (+ + − + + + −). Three major haplotypes based on Orkin et al. [19] III (60.4%), V (24.0%), and IV (9.7%) were accounted for the majority of HbE alleles and they were linked with *Xmn* I +, −, and +, respectively. Therefore only 71.43% of HbE allele is linked with the T allele (or + genotype) of *Xmn* I. Only the frameworks 2 (+ −) and 3 (− +) based on Antonarakis et al. [13] were found, while the framework associated with (−−) or (+ +) patterns were not observed. The summary of all eight *β* globin haplotypes is shown in Table 1.

**Figure 2:**
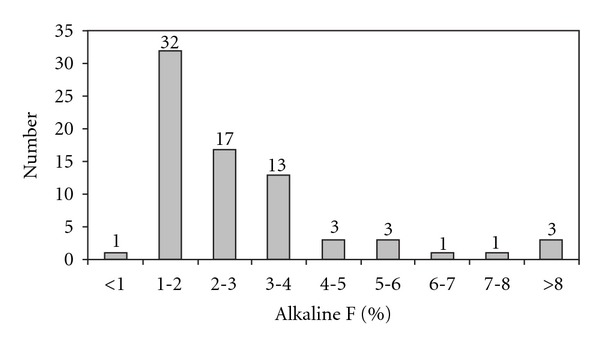
Hemoglobin F distribution in 74 individuals with homozygous Hb E.

Seventy-four individuals who had all complete hematological and hemoglobin data with molecular results including Hb E genotypes and their linked *β* globin haplotypes were subjects for the further association analyses. Detailed hematological and clinical data of these individuals have been described previously [14]. In summary, majority of homozygous Hb E cases have borderline hemoglobin (Hb) and hematocrit (Hct) levels with significantly low mean corpuscular volume (MCV 58 ± 5.2 fL). The levels of baseline hemoglobin were highest in male subjects (12.6 ± 1 g/dL) followed by females (11.4 ± 1 g/dL), and pediatric cases (10.6 ± 0.8 g/dL). None of these individuals had hepatosplenomegaly and require blood transfusion [14]. At the beginning of the study, 12 individuals were identified with high Hb F over 5% at their steady stage. During our follow-up, Hb F levels in 4 pediatric cases (under 15 years) decreased or were absent. This finding suggests that increase of Hb F levels in some pediatric individuals with homozygous Hb E might be resulted from persistent postnatal production of *γ* globin expression in which declined later on in their late childhood [6]. While in adult cases, their Hb F levels remained persistently high excluding the possibility of temporary hematopoietic stress that might affect the expression of the *γ* globin genes. These eight individuals with homozygous Hb E (10.8%) had high level of Hb F (average 10.5%, range 5.8–14.3%), while the rest had Hb F less than 5%. Distribution of steady stage Hb F percentages in all 74 homozygous Hb E is shown in Figure 2. Nevertheless, female individuals seemed to have significantly higher average Hb F determined by alkaline Hb F measurement than their male counterparts (*P* < 0.05, Table 2). This finding was consistent with previous studies in the general population that higher Hb F has been observed in female leading to the comprehensive studies on quantitative-trait locus (QTL), including X-linked gene on Xp22.2, for the expression of *γ* globin gene [21–23]. However the main causative mechanism underlying sexual difference remains obscure. Interestingly, hematological data of these homozygous Hb E cases with high Hb F are not different from those who had less Hb F or none (data not shown). 

Interestingly, there was no significant association between specific *β* globin haplotypes and *Xmn* I polymorphism in these eight individuals with high Hb F compared to the rest. Moreover, we failed to identify any association between eight *β* globin haplotypes and the baseline Hb F levels determined by alkaline denaturation in all 74 cases (data not shown). Even though the average alkali F levels were albeit increased in homozygous Hb E with *Xmn* I (+ +) compared to (+ −) it was statistically insignificant within the same sex (Table 2).

## 4. Discussion

To the best of our knowledge, this study, herein, provides the most extensive analyses of the *β* globin haplotypes linked to Hb E alleles to date. Due to a higher number of individuals with homozygous Hb E used in this study, we found that the haplotypes linked to Hb E in Thailand might be more heterogeneous than was previously thought. At least 8 different haplotypes including two novel ones were found in this study; however, in the majority of these cases (94.3%), Hb E alleles were linked with the three major haplotypes; (a) (III), (b) (V), and (c) (IV), consisting with previous studies in our population and supporting the hypothesis of multiple origins of Hb E in this part of the world [11–13]. In Bangkok, where it is a melting pot of the whole country due to a massive immigration of people from different parts of Thailand, it is not simple to trace and track back their original regions. Therefore it is not surprising that we found such a heterogeneity including two new haplotypes in Thailand: Th1; (− − − − − + −) and Arab-Indian; (+ + − + + + −). The latter is identical to *β* ^S^ haplotype reported from previous studies [20, 24]. However, these two new haplotypes were closely similar to the two common haplotypes of b (V) and a (III), respectively. Only the specific restriction site at the *ε*-*Hind* II site is different suggesting that these two novel haplotypes may arise from interallelic gene conversion than an independent origin [25]. Moreover our study also confirms the previous construction of the *β* globin haplotypes in other studies which had a limited number of homozygous condition and used the haplotype analyses mainly from Hb E heterozygotes [11, 12].

In this study, we further addressed the correlation between the *β* globin haplotype (implying *cis*-regulatory sequences) and the predisposition of Hb F production. We found several individuals with homozygous Hb E and high levels of Hb F in which other extrinsic factors such as intercurrent infection, hematopoietic stress, and erythropoiesis could not be accounted for. These individuals may provide a novel, rather less complicated natural model for further study on molecular mechanism controlling *γ* globin expression as they all have identical *β* globin gene defects (Hb E) and their linked *β* globin haplotypes could be easily constructed. However, we did not succeed to identify any positive correlation, even with the *Xmn* I polymorphism suggesting that Hb F production found in these homozygous Hb E individuals might be mainly controlled by *trans*-acting mechanism(s) including HBS1L and BCL11A [6, 26]. On the contrary, the strong association between the extensive *β* globin haplotypes and clinical severity of Hb E/*β* thalassemia patients were found in Thai patients and this was accounted by a link between the probable functional SNP, *Xmn* I, and the propensity to increase Hb F production [10]. It is possible that the levels of erythropoietic stress and/or globin imbalance found in our homozygous Hb E individuals must be significantly lesser compared to Hb E/*β* thalassemia and this might not be enough to trigger the switching of postnatal *γ* globin expression as the Hb E/*β* thalassemia syndrome does. Interestingly, our study showed that Hb E alleles in our study population are linked to both T (+) and C (−) alleles of the *Xmn* I site suggesting a high heterogeneity of Hb E alleles within Thai population and highlighting a great consideration for future determination of haplotype and SNPs, which links to the phenotypes related to Hb F and clinical severity.

In conclusion, the association of Hb F production with the *Xmn* I and the *β* globin haplotypes as a marker could not be confirmed using homozygous Hb E condition. However, this might suggest a limitation of the model used in this study as the influence of *cis*-regulatory elements might not be strong enough to be detected under a condition with slighter hematopoietic stress such as homozygous Hb E. 

## Figures and Tables

**Table 1:** Summary of 8 different *β* globin haplotypes linked to Hb E alleles from 154 Hb E chromosomes.

Number	*β*-globin haplotypes								*Xmn* I	Total *N* = 154 (%)
	Annotations	*εHin*dII	G_*γ*_ *Hin*dIII	A_*γ*_ *Hin*dIII	5′*βφHin*dII	3′*βφHin*dII	5′*β* AvaII	3′*βHin*fI		
(1)	(a) or III	−	**+**	−	**+**	**+**	**+**	−	**+**	93 (60.39)
(2)	(b) or V	**+**	−	−	−	−	**+**	−	−	37 (24.02)
(3)	(c) or IV	−	**+**	−	**+**	**+**	−	**+**	**+**	15 (9.74)
(4)	Y1	−	**+**	−	−	**+**	**+**	−	−	4 (2.60)
(5)	VIII	−	**+**	−	**+**	−	**+**	−	**+**	1 (0.65)
(6)	Y2	−	**+**	**+**	−	**+**	**+**	−	−	2 (1.30)
(7)	Th-1	−	−	−	−	−	**+**	−	−	1 (0.65)
(8)	Arab-Indian	**+**	**+**	−	**+**	**+**	**+**	−	**+**	1 (0.65)

(a), (b), and (c) were based on Antonarakis et al., 1982 [13]. III, V, IV, and VIII were based on Orkin et al., 1982 [19]. Y1 and Y2 were based on Yongvanit et al., 1989 [12]. Th-1 is a novel *β* globin haplotype found in this study. Arab-Indian haplotype was based on Nagel and Ranney, 1990 [20].

**Table 2:** Comparison of alkaline F % (±SD) in homozygous HbE with different *Xmn* I alleles.

*Xmn* I genotype	Male		Female		*P* value
	*N*	Alkaline F (%)	*N*	Alkaline F (%)
+/+	18	2.34 ± 1.24	24	3.38 ± 0.26	<0.05
+/−	10	1.93 ± 0.27	20	2.83 ± 2.5	<0.05
−/−	2	1, 3.5	0	NA	NA

*significantly difference (*P* < 0.05) between male and female subjects with similar *Xmn*I genotypes. NA: not available.
